# Leveraging tuberculosis case relative locations to enhance case detection and linkage to care in Swaziland

**DOI:** 10.1186/s41256-018-0058-y

**Published:** 2018-02-05

**Authors:** Marie Brunetti, Sathyanath Rajasekharan, Piluca Ustero, Katherine Ngo, Welile Sikhondze, Buli Mzileni, Anna Mandalakas, Alexander W. Kay

**Affiliations:** 1 Clinton Health Access Initiative, Mbabane, Swaziland; 2 Global TB Program, Department of Pediatrics, Baylor College of Medicine, Baylor International Pediatric AIDS Initiative, PO Box 110, Mbabane, Swaziland; 3 Swaziland National Tuberculosis Control Program, Mbabane, Swaziland; 4 Global TB Program, Baylor Children’s Foundation-Swaziland, Mbabane, Swaziland

**Keywords:** Mapping, Geospatial, Relative location, Active case finding

## Abstract

**Background:**

In Swaziland, as in many high HIV/TB burden settings, there is not information available regarding the household location of TB cases for identifying areas of increased TB incidence, limiting the development of targeted interventions. Data from “Butimba”, a TB REACH active case finding project, was re-analyzed to provide insight into the location of TB cases surrounding Mbabane, Swaziland.

**Objective:**

The project aimed to identify geographical areas with high TB burdens to inform active case finding efforts.

**Methods:**

Butimba implemented household contact tracing; obtaining landmark based, informal directions, to index case homes, defined here as relative locations. The relative locations were matched to census enumeration areas (known location reference areas) using the Microsoft Excel Fuzzy Lookup function. Of 403 relative locations, an enumeration area reference was detected in 388 (96%). TB cases in each census enumeration area and the active case finders in each Tinkhundla, a local governmental region, were mapped using the geographic information system, QGIS 2.16.

**Results:**

Urban Tinkhundla predictably accounted for most cases; however, after adjusting for population, the highest density of cases was found in rural Tinkhundla. There was no correlation between the number of active case finders currently assigned to the 7 Tinkhundla surrounding Mbabane and the total number of TB cases (Spearman rho = −0.57, *p* = 0.17) or the population adjusted TB cases (Spearman rho = 0.14, *p* = 0.75) per Tinkhundla.

**Discussion:**

Reducing TB incidence in high-burden settings demands novel analytic approaches to study TB case locations. We demonstrated the feasibility of linking relative locations to more precise geographical areas, enabling data-driven guidance for National Tuberculosis Programs’ resource allocation. In collaboration with the Swazi National Tuberculosis Control Program, this analysis highlighted opportunities to better align the active case finding national strategy with the TB disease burden.

## Background

Swaziland has an estimated TB incidence of 565 cases/100,000 persons and TB/HIV co-infection rates above 70% in 2015 [1]. As in many high HIV/TB burden settings there is not information regarding the geo-spatial distribution of TB cases. Obtaining this information is hampered by several elements; the lack of registered addresses for households, a paper based registration system, and the fact that TB cases are classified by the location of the clinic where the patient is diagnosed vs. the patient’s residence. Therefore, without the labor and cost intensive step of obtaining Geo-positioning System (GPS) coordinates of all index case residences, there is often little data for the National TB Program (NTP) to use to localize TB cases apart from the clinic or region in which they choose to access care.

The World Health Organization (WHO) 2016 Global Tuberculosis report emphasizes the importance of obtaining TB case location data. Defining TB hotspots enables effective allocation of NTP resources [1]. Increased TB transmission has been linked to areas of high population density, poverty and HIV [2–4]. In Swaziland, malaria elimination projects have had success mapping areas of transmission contributing to reduced malaria incidence [5]. Recent modeling studies of tuberculosis in Rio De Janeiro suggest that controlling a TB hot-spot can have a significant and disproportionate impact on overall TB control in a community as a whole [6]. While identifying and controlling hotspots of TB transmission is a well established method of TB control programs in low-burden, high-resource settings [7, 8], it is not inherently clear that the method would have the same impact in TB high-burden settings. Nevertheless, strategies to identify and control TB hot-spots are increasingly being implemented in resource-constrained high TB burden settings [9].

GPS coordinates are the gold standard with regard to defining case locations, but in settings without registered addresses this requires visiting each TB case household to collect coordinates [10]. In many settings, available resources do not support this approach. Additionally, household addresses in Swaziland are generally unregistered, and are typically described using local landmarks, further impeding documentation of case locations. As such, here we describe a novel method to systematically generate TB case location data from informal, landmark based, directions to patient homes with the intention of informing TB control activities.

## Methods

Household location data for TB cases collected by “Butimba”, a Stop TB Partnership-TB REACH active case finding project conducting household contact tracing, was re-purposed to provide insight into the location of TB cases surrounding Mbabane, Swaziland [11]. From March 2013 to November 2015, Butimba enrolled 3258 TB cases in three of four regions in Swaziland [11]. Butimba implemented household contact tracing for index cases and often obtained landmark based, informal directions to index cases homes, defined here as a relative location.

In 2016, the Swaziland National Tuberculosis Control Program (NTCP) employed 369 TB active case finders (ACFs), distributed by Tinkhundla, a geographic designation based on local community governments. ACFs are lay people trained to visit households affected by tuberculosis in order to identify and refer TB contacts to health facilities as indicated by local guidelines. Unfortunately, no TB case location data was available to inform ACF geographic placements as current TB cases are registered to one of four regions in Swaziland, but are not localized further to the household or community level [12]. Therefore, we analyzed household relative locations generated through the Butimba project for TB cases treated at two Basic Management Units (BMUs) in Mbabane, Swaziland. This pilot project aimed to identify smaller geographical areas with high total and population adjusted TB burdens to inform local TB epidemiology, guide NTCP’s ACF distribution, and improve the cost-efficiency of national case finding interventions.

All TB cases at the two large BMUs from the Butimba database, Baylor Clinic and Mbabane Government Hospital, were identified a priori to inform case distribution surrounding Mbabane. The contact tracing forms for all index cases were reviewed for the presence of relative locations. Relative locations were then matched to a reference list of census enumeration areas (EAs) using the Microsoft Excel Fuzzy Lookup function (Fuzzy Lookup v1.3.0, Redmond, Washington, USA) that quickly identifies and matches data records that are not spelled exactly the same but are textually similar as defined by a Jaccard similarity index [13]. All results were manually reviewed for errors, with local experts applying additional attention on all cases with a Jaccard similarity index of <0.8, meaning that the probability that there is a textual match is under 80%. Relative locations that failed to match to EAs using Fuzzy Lookup were manually matched by local experts.

**Fig. 1:**
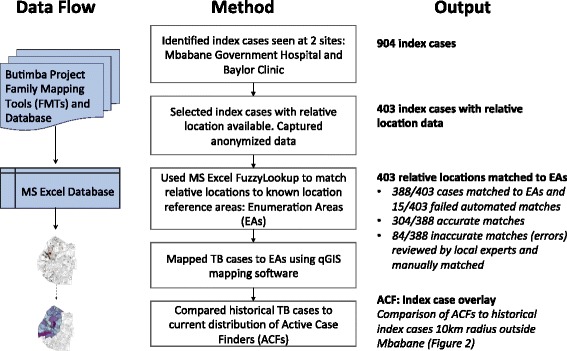
Process algorithm outlining the analysis of TB case relative locations

**Fig. 2:**
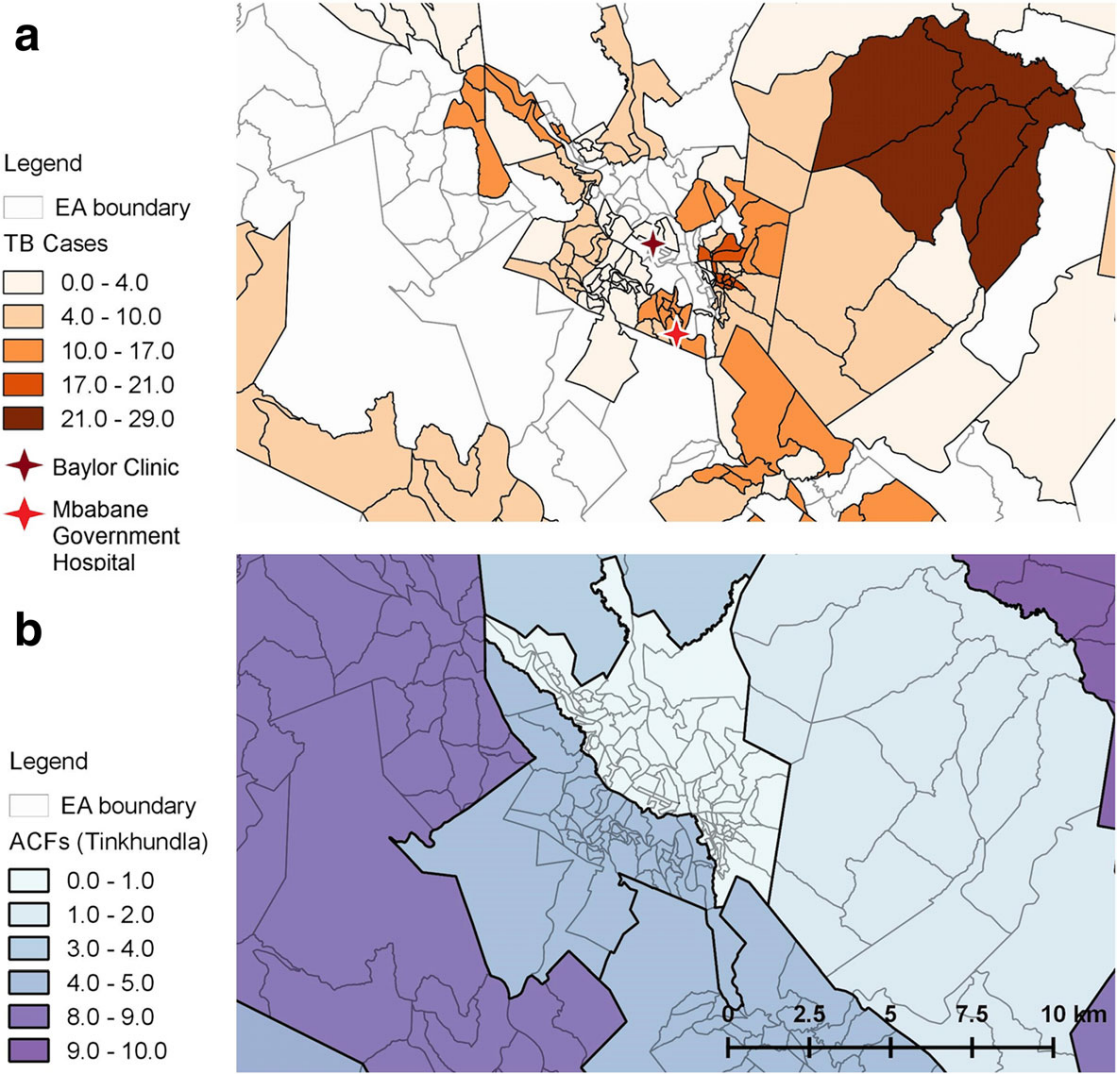
QGIS map of Mbabane and an approximate 10 km radius depicting TB cases per EA and ACFs per Tinkhundla. **a** Total TB cases during identified during the Butimba project period per census EA. **b** Total ACFs assigned to each Tinkhundla. Tinkhundla comprise many EAs and are designated by the bold borders

After linking the index cases homestead locations to an EA, the absolute TB case number and the population (2007 census) adjusted case number were defined for each EA. Census EAs are sub-regions of Tinkhundlas, and the absolute TB case number and adjusted TB case numbers were also applied to each Tinkhundla. Total TB cases and population adjusted TB cases in each EA were mapped using the geographic information system, QGIS 2.16 (QGIS Development Team, 2016. QGIS Geographic Information System. Open Source Geospatial Foundation. https://www.qgis.org/en/site/). QGIS was also used to map active case finders assigned to each Tinkhundla. The analytic pathway is outlined in Fig. 1 and a visual representation of ACF designations versus total TB cases was created using qGIS (Fig. 2a, b). Correlation analysis between ACF placements and TB cases were performed using Spearman’s Rank-Order Correlation and performed using R Version 3.3.2 (R: A language and environment for statistical computing. R Foundation for Statistical Computing, Vienna, Austria. URL https://www.R-project.org/.)

## Results

A total of 213 TB cases at Baylor Clinic and 691 TB cases at Mbabane Government Hospital were identified to inform case distribution surrounding Mbabane, and after reviewing all index case forms, relative location information was present for 45% (403/904). Of 403 relative locations, an EA reference was detected in 96% (388/403). 4% (15/403) failed to match but were successfully manually mapped by local experts. Overall, 22% of matches (84/388) had a Jaccard Similarity Index of <0.8 meaning that the probability that there was a match was under 80%. Of these 84 results, 45 errors were easily identifiable due to a mismatch between the text and the EA name. 39 were erroneous automated matches, which were then matched to an EA by a local expert. Reasons for missed identification included major spelling differences, the presence of multiple locations, or individuals’ names within the records that closely matched EA names.

After linking the index case homestead locations to an EA, the absolute case number and the population (2007 census) adjusted case number were defined for each EA and Tinkhundla. 95% (383/403) of the cases were located in 7 Tinkhundla of varying population density surrounding Mbabane. The population was predominantly rural in 4/7 Tinkhundla and predominantly urban in 3/7. The average annual population adjusted cases for the 7 Tinkhundla was 160 TB cases/100,000 persons, 28% (160/565) of the national population adjusted estimate, indicating our sample contained approximately a quarter of all cases in the area.

**Fig. 3:**
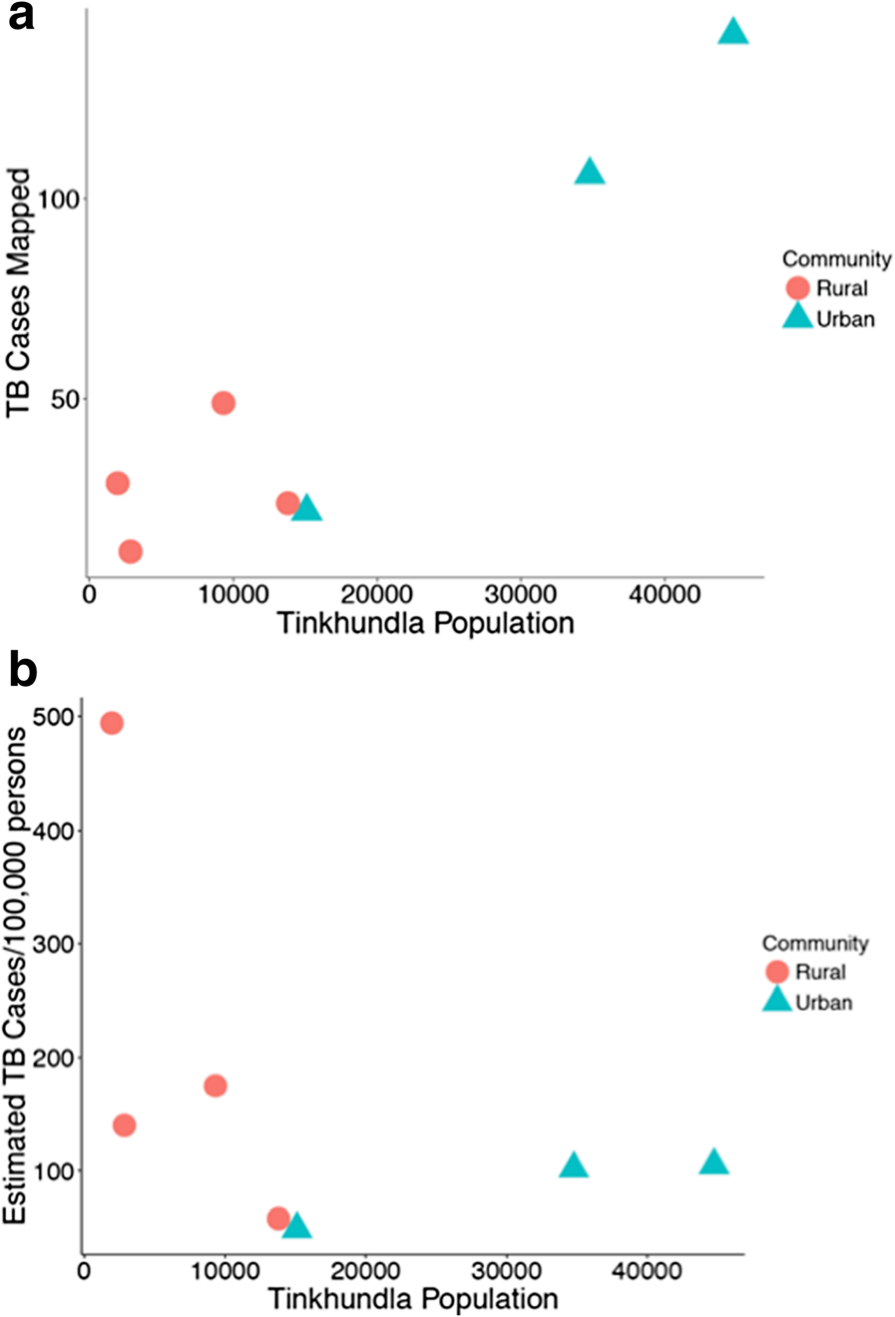
Scatter plots defining the total cases mapped (**a**) and the annual population adjusted case number of TB cases (**b**) per 7 Tinkhundla surrounding Mbabane as a function of population. Tinkhundla are dichotomized as predominantly urban vs. rural

**Fig. 4:**
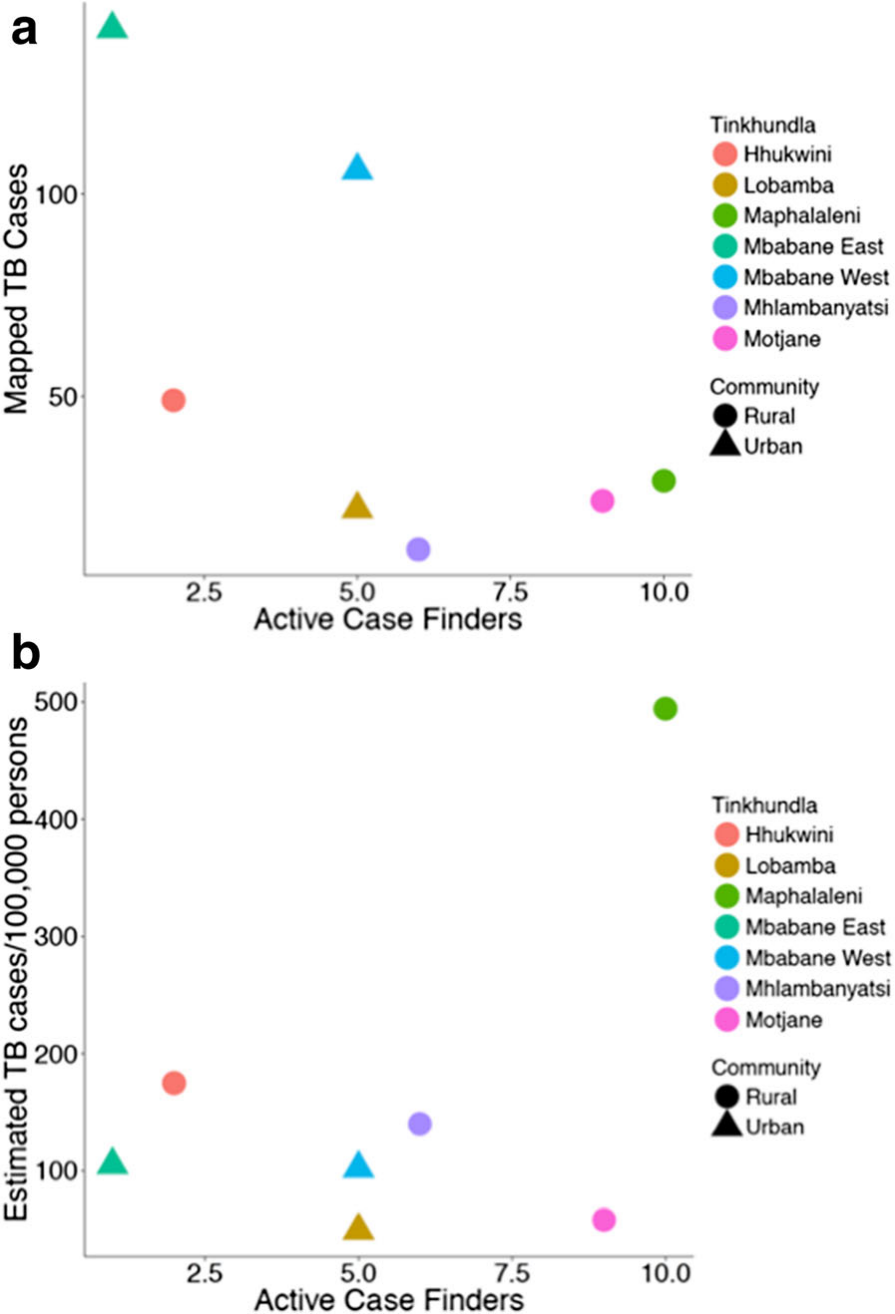
Scatter plots defining the total cases mapped (**a**) and the annual population adjusted case number of TB cases (**b**) per each of 7 Tinkhundla surrounding Mbabane by Active Case Finder (ACF) placements per Tinkhundla. Tinkhundla are color coded and also depicted as predominantly urban vs. rural

Urban, high-population, Tinkhundla predictably accounted for most cases; however, after adjusting for population density the highest population adjusted TB incidence was found in rural Tinkhundla (Fig. 3a, b). ACFs were originally assigned without analysis of the TB case location data and no correlation was identified between the number of ACFs assigned to 7 Tinkhundla surrounding Mbabane and the total number of TB cases (Spearman rho = −0.57, *p* = 0.17) or the population adjusted TB cases (Spearman rho = 0.14, *p* = 0.75) per Tinkhundla (Fig. 4a, b).

## Discussion

Here we report on a novel method to analyze the relative locations of TB cases in order to enhance TB control activities. Despite the informal appearance of these relative locations they could be digitalized and linked to known location reference areas, in this case census EAs. The location data was extracted with a high degree of accuracy using an automated approach, reducing the time and resources required to manually analyze relative location data on a large scale. This analysis identified TB hotspots surrounding Mbabane that were previously unknown. While most cases were identified, as expected in urban, densely populated areas, the highest population adjusted incidence of cases occurred in rural areas.

The value of localizing TB cases to inform TB control efforts is well established. There is robust data demonstrating the utility of mapping TB cases based on household GPS coordinates to define TB epidemiology [2, 10]. In addition, TB case location data at the provincial or city level has also been displayed through qGIS software to inform trends in TB spread overtime [14]. This report is unique as our pragmatic and feasible methods capitalized on relative locations to define TB case locations surrounding Mbabane, Swaziland. This approach of analyzing relative household locations has not previously been described to inform TB epidemiology or guide deployment of TB control resources such as active case finders.

This data showed that ACF placements did not statistically correlate with total TB case burden or population adjusted TB cases. However, as a whole, more ACFs were assigned to rural regions with a low total case burden but a high population adjusted TB incidence. Certainly, more robust TB control efforts in areas with a high population adjusted TB case load but low overall population may have a profound impact on the number of future TB cases in those areas. The argument could also be made that more ACFs would be better utilized in areas with the highest number of overall TB cases. A number of other regional factors such as topography, rates of mine workers in the community, the prevalence of co-morbid conditions such as HIV and socioeconomic status also need to inform TB control efforts, but the data derived from TB case relative locations provides an additional tool for NTCPs to enhance TB control efforts in high-burden settings.

Although feasible in our resource constrained TB high burden setting, our pilot project has several limitations. The analyzed cases represented only a portion of the total TB cases in Mbabane during the project period, and may not be representative of all cases. Additionally, the homestead assignment to an EA is an estimated location. GPS coordinates are more precise but such datasets are not widely available; however, the increasing prevalence of GPS enabled mobile phones may make obtaining GPS coordinates more practical in the future [15]. Lastly, this data was analyzed retrospectively, potentially missing recent changes in TB epidemiology. However, relative household location data could be collected on all index cases prospectively, allowing for annual updates of TB incidence in EAs and Tinkhundlas throughout Swaziland. Despite limitations, our pragmatic approach demonstrated proof of concept for the value in analyzing relative locations to inform TB control activities. Extraction of more precise location data from relative location data is an area of scientific interest [16], but to our knowledge has not previously been applied to a public health context or to TB control efforts.

## Conclusion

The End TB strategy calls for a 90% reduction in TB incidence rate by 2035 [17], a goal that demands novel approaches to improve active case finding in high-burden settings. This project demonstrated the feasibility of linking patient’s household relative locations to more precise geographical areas in areas where GPS mapping is still not feasible. Our results were employed by the Swaziland NTCP to enrich their information regarding local TB epidemiology and to inform resource allocation. Our automated methods were not labor intensive and suggest that the project could be replicated in different settings and without significant human resource investments. Hence, our method may provide a feasible alternative to GPS mapping that can still provide information to support a data-driven approach to NTCP resource allocation and TB control activities in TB high burden settings similar to Swaziland.

## References

[1] World Health Organization. Global Tuberculosis Report 2016. Geneva: WHO Press; 2016.

[2] Munch Z, Van Lill SWP, Booysen CN, Zietsman HL, Enarson DA, Beyers N (2003). Tuberculosis transmission patterns in a high-incidence area: a spatial analysis. Int J Tuberc Lung Dis.

[3] Spence DP, Hotchkiss J, Williams CS, Davies PD. Tuberculosis and poverty. BMJ. 1993;307:759–61. 10.1136/bmj.307.6907.759 PMC1696420 8219945

[4] Chaisson RE, Martinson NA. Tuberculosis in Africa—combating an HIV-driven crisis. N Engl J Med. 2008;358:1089–92. 10.1056/NEJMp0800809 18337598

[5] Reiner RC Jr, Le Menach A, Kunene S. Mapping residual transmission for malaria elimination. elife. 2015;4:1–16. 10.7554/eLife.09520 PMC4744184 26714110

[6] Dowdy DW, Golub JE, Chaisson RE (2012). Heterogeneity in tuberculosis transmission and the role of geographic hotspots in propagating epidemics.

[7] Roth D, Otterstatter M, Wong J, Cook V, Johnston J, Mak S (2016). Identification of spatial and cohort clustering of tuberculosis using surveillance data from British Columbia, Canada, 1990-2013. Soc Sci Med.

[8] Goswami ND, Hecker EJ, Vickery C, Ahearn MA, Cox GM, Holland DP, Naggie S, Piedrahita C, Mosher A, Torres Y, Norton BL, Suchindran S, Park PH, Turner D, Stout JE (2012). Geographic information system-based screening for TB, HIV, and syphilis (GIS-THIS): a cross-sectional study. PLoS One.

[9] Yeboah-Manu D, Asare P, Asante-Poku A, Otchere ID, Osei-Wusu S, Danso E, Forson A, Koram KA, Gagneux S (2016). Spatio-temporal distribution of mycobacterium tuberculosis complex strains in Ghana. PLoS One.

[10] Shah L, Choi HW, Berrang-Ford L, Henostroza G, Krapp F, Zamudio C, Heymann SJ, Kaufman JS, Ciampi A, Seas C, Gotuzzo E, Brewer TF (2014). Geographic predictors of primary multidrug-resistant tuberculosis cases in an endemic area of lima, Peru. Int J Tuberc Lung Dis.

[11] Mandalakas AM, Ngo K, Alonso Ustero P, Golin R, Anabwani F, Mzileni B, Sikhondze W, Stevens R (2017). BUTIMBA: intensifying the hunt for child TB in Swaziland through household contact tracing. PLoS One.

[12] Kingdom of Swaziland, Ministry of Health. Swaziland Annual National Tuberculosis Control Program Report, 2015. Published 2016:1–36.

[13] Ganjam K. Microsoft Fuzzy Lookup Add-In for Excel. 2011:1–4. Available from https://atidan.files.wordpress.com/2013/08/fuzzy-lookup-add-in-for-excel.pdf. Accessed 14 Nov 2017.

[14] Gurjav U, Burneebaatar B, Narmandakh E, Tumenbayar O, Ochirbat B, Hill-Cawthorne GA, Marais BJ, Sintchenko V (2015). Spatiotemporal evidence for cross-border spread of MDR-TB along the trans-Siberian railway line. Int J Tuberc Lung Dis.

[15] Denkinger CM, Grenier J, Stratis AK, Akkihal A, Pant-Pai N, Pai M (2013). Mobile health to improve tuberculosis care and control: a call worth making [review article]. Int J Tuberc Lung Dis.

[16] Zhang X, Mitra P, Klippel A (2011). Identifying destinations automatically from human generated route directions.

[17] World Health Organization (2016). The end TB strategy: global strategy and targets for tuberculosis prevention, care and control after 2015.

